# Modelling droplet size distribution in inline electrostatic coalescers for improved crude oil processing

**DOI:** 10.1038/s41598-023-46251-4

**Published:** 2023-11-18

**Authors:** Ghazal Kooti, Bahram Dabir, Reza Taherdangkoo, Christoph Butscher

**Affiliations:** 1 Department of Petroleum Engineering, Amirkabir University of Technology, Tehran, Iran; 2 Chair of Engineering Geology and Environmental Geotechnics, TU Bergakademie Freiberg, Freiberg, Germany; 3 Department of Chemical Engineering, Amirkabir University of Technology, Tehran, Iran

**Keywords:** Chemical engineering, Colloids, Computational science

## Abstract

Water-in-oil emulsions pose significant challenges in the petroleum and chemical industrial processes, necessitating the coalescence enhancement of dispersed water droplets in emulsified oils. This study develops a mathematical model to predict the evolution of water droplet size distribution in inline electrostatic coalescers (IEC) as a promising means to improve the water separation efficiency of current oil processing systems. The proposed model utilizes the population balance modelling approach to effectively simulate the dynamic and complex processes of coalescence and breakage of droplets in crude oil which directly influence the separation efficiency of the process. The method of classes as an effective mathematical technique is selected to solve the population balance equation (PBE). The accuracy of the model and considered assumptions agree well with experimental data collected from the literature. The results demonstrate the model's ability to accurately simulate droplet coalescence and breakage in emulsified oil while predicting droplet size distribution and water removal efficiency. The electric field strength, residence time, and fluid flow rate significantly influence the coalescence of droplets. At 4 kV and 5 m^3^/h after 4 s the mean diameter of droplets (D_50_) and separation efficiency reach the maximum of 94.3% and 432 µm, respectively. The model enables the optimization of operational conditions, resulting in increased performance and reliability of oil-processing systems while reducing the energy consumption and use of chemical demulsifiers. Additionally, utilization of the device in optimized conditions significantly reduces the size and weight of downstream separation equipment, which is particularly advantageous for heavy oils and offshore fields.

## Introduction

Crude oil naturally contains a significant amount of water, thus water-in-oil emulsions are easily formed during the extraction, storage, transportation, and processing of crude oil. The presence of natural surfactants in the oil, such as asphaltenes, resins, naphthenic acids, and fine solids, could contribute to the stability of these emulsions^1^. The removal of water from crude oil is crucial to avoid corrosion of the process equipment, catalyst poisoning in downstream refineries, and extra transportation costs owing to increased volume as well as increased viscosity^2^. Therefore, the effective elimination of a dispersed water phase from a continuous oil phase is significant.

The largest water drops are removed through gravitational separation, but the required time for separation of small water droplets with a diameter of less than 50 µm is long and inefficient^3,4^. Available methods to address this challenge include chemical demulsification^4^, gravity or centrifugal settling^5^, pH adjustment, filtration, heat treatment, membrane separation, and electrostatic demulsification^6^. Each of these techniques offers distinct advantages, making them viable options for achieving the desired phase separation in specific contexts within the realm of chemical processes. Considering high efficiency, electrical demulsification is regarded as the most favourable method in the petroleum industry among the options mentioned above^7^.

External electric fields have been widely utilized in the separation of water-in-oil emulsions^8^. This technique originated from the study of Cottrell and Speed^9^, which primarily focused on dehydrating crude oils and the desalting process. The classical technique involves using a relatively weak electric field (approximately 500 V/cm) to the fluid mixture at rest or in slow motion^3^. Electrostatic treaters use high voltage alternating current (AC) fields, or to a lesser extent direct current (DC) fields, to promote the coalescence of water droplets in crude oil to improve the phase separation^10–12^. Conventional electro-separators tend to be large in size due to the extended residence times required for the effective separation of enlarged water droplets from crude oil in the electro-coalescence zones and settling regions. More recent advancement in electro-coalescer technology, known as inline electrostatic coalescers (IEC), has led to significant improvement in water separation efficiency. In this apparatus, the water/oil mixture flows in a pipe equipped with a series of insulated active and grounded electrodes and is subjected to an AC electric field ranging from 1 to 4 kV. In these conditions, the mean diameter of the droplets (initially around 30 µm) is magnified by a factor of 10 approximately. This indicates a significant coalescence rate^13^*.*

Previous studies have focused on modelling the conventional electro-coalescence process. Chiesa^14^ conducted a study on a stagnant water-in-oil emulsion exposed to an external AC electrical field, and compared simulations and optical observations which showed satisfactory agreement for low water volume fractions. Al-Otaibi et al.^15^ presented a flow diagram of a typical crude oil dehydration plant. They utilized an artificial neural network to simulate and optimize the process. Melheim and Chiesa^16^ studied the mutual interaction between turbulence and the electric field. To investigate this phenomenon, they employed a numerical framework based on the Eulerian–Lagrangian approach. Alves and Oliveira^17^ developed a semi-empirical mathematical model that correlates the operational and free variables to response variables. Using data collected from their pilot plant test on various crude oils, they calculated the constants of their model to validate and fine-tune its accuracy. Furthermore, Bresciani et al.^18^ employed a stochastic method to implement the sequence of droplet collisions. Their model involved calculating the velocity of water droplets by applying a force balance on a pair of drops. Following this, the concept of cellular automata was utilized to simulate the entire group of droplets and determine the separation of water from oil emulsions. Meidanshahi et al.^19^ utilized a bivariate PBE to model the steady-state distribution of water droplet sizes and salt concentration along an AC conventional electrostatic desalting vessel. They examined both one-stage and two-stage processes. To validate their model, they compared it with pilot plant data. Aryafard et al.^20^ expanded on the work of Meidanshahi et al.^19^ and investigated the effect of electric field strength in an industrial two-stage crude oil desalting process using a numerical method. Khajehesamedini et al.^21^ used PBE and developed coalescence and capture kernels for the electro-coalescence process based on batch experiments. Ranaee et al.^22^ employed artificial intelligence and evaluated the performance of a crude-oil desalting/demulsification system with a combined use of global sensitivity analysis (GSA), machine learning, and rigorous model discrimination criteria. However, in previous studies, the occurrence of droplet breakage has not been considered in modelling the process of electro-coalescence. This phenomenon can take place simultaneously and impacts the efficiency of water separation. Moreover, only few studies focused on the modelling of inline electrostatic devices, including the work by Melheim and Chiesa^16^ which have solely concentrated on droplet coalescence. Considering the increasing demand to address flow conditioning challenges in the dehydration process, especially in offshore platforms with limited space^23^, and the need to enhance the efficiency of heavy oil processing systems, a comprehensive understanding and modelling of modern inline electrostatic coalescers (IEC) become crucial.

**Figure 1:**
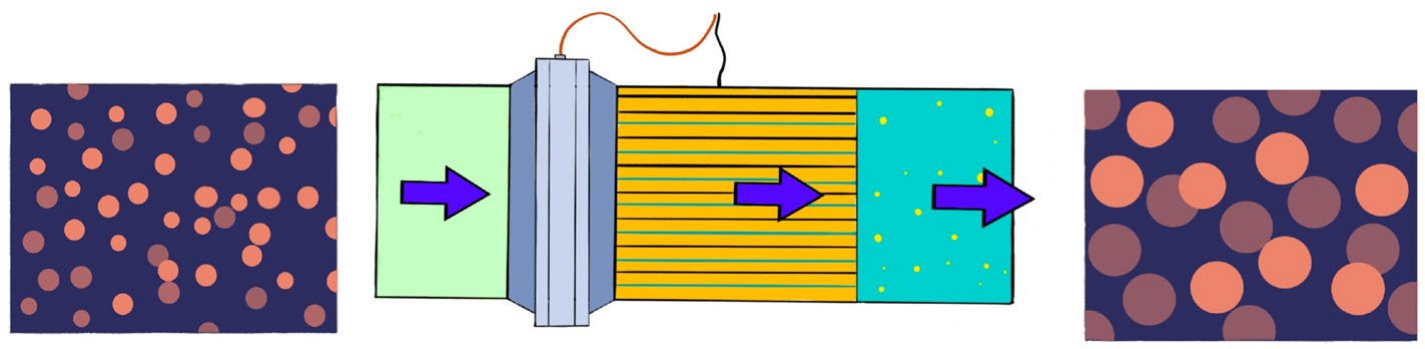
A schematic presentation of an inline electrostatic coalescer device (modified after Grave et al.^13^).

IEC is a pipe-based device equipped with a series of insulated active and grounded electrodes which is exposed to an AC electric field ranging from 1 to 4 kV/cm^13^ (Fig. 1). This effective system enhances the separation of water from crude oil by destabilizing oil-continuous emulsion and promotes a shift in the size distribution towards larger water droplets. The electric field causes water droplets that are dispersed in the oil phase to become polarized and collide in a moderately turbulent flow regime. The polarized drops are provided with a strong-range attraction force that enables them to break the interfacial film between them and eventually merge to create a larger droplet^12,24^.

This device achieves significant increases in droplet size at short residence times (e.g. 4 s), and provides a compact alternative to standard industrial electro-coalescers. By average, the IEC is capable of magnifying droplet size by a factor of 10 and may also be employed as a standalone tool or in combination with other equipment to enhance the efficiency of existing separation systems and improve process bottlenecks^13^.

Typically, the separation of water from crude oil upstream involves multi-stage separation vessels, and the size of these separation trains may pose limitations for use in offshore platforms and subsea separation applications, where the benefits of utilizing this compact device are more pronounced^25^.

The inline electrostatic coalescer is not a separator itself. The function of the coalescer is to increase water-in-oil droplet sizes and break emulsions. This allows for efficient separation in a downstream separator. The downstream separator can be a gravity separator, a pipe separator, or a cyclonic liquid–liquid separator. IEC is distinguished by moderate turbulence that induces frequent collisions among water droplets and is further aided by an electrical field that stabilizes the film between the colliding droplets. Therefore, the probability of droplet coalescence is increased^13^.

This study aims to develop a model that accounts for both coalescence and breakage of emulsion droplets under a static electric field within an IEC device, using a population balance approach. The effects of the strength of the electric field, inlet flow rate, and residence time on the droplet size distribution and separation efficiency are discussed to guide the selection and setting of the optimal operating parameters of the device. Furthermore, the accuracy of the model and assumptions is evaluated using performance tests results published in the literature.

## Fundamental mechanisms of electro-coalescence

The process of electro-coalescence between two water droplets can be explained through three distinct stages: (1) the droplets approach each other by overcoming long-range flocculation forces, (2) the film separating the droplets undergoes thinning or drainage, thereby reducing the interfacial area, and (3) once the film reaches a critical thickness, any disruption or instability leads to its rupture or breakdown, ultimately resulting in the coalescence of the water droplets^11^.

In order to maximize the water separation performance, the electrostatic forces must be capable of promoting droplet coalescence to diameters greater than Stokes diameter^26^.

1 $${d}_{stokes}={\left(\frac{18{\mu }_{c}{u}_{s}}{g\left({\rho }_{d}-{\rho }_{c}\right)}\right)}^{0.5},$$

where ${\mu }_{c}$, ${u}_{s}$, ${\rho }_{d}$, ${\rho }_{c}$ and $g$ respectively represent dynamic viscosity of the continuous phase, relative velocity of the dispersed droplets through the continuous phase, density of the dispersed phase, density of the continuous phase, and constant of gravitational acceleration.

The three electrostatic forces are the dipolar, electrophoretic, and dielectrophoretic forces.

### Dipolar force

When two induced dipoles come into proximity, the droplets are subjected to each other's non-uniform field, leading to possible attraction or repulsion. Regarding the calculation of dipolar force (F_dip_), three models have been proposed so far: the point-dipole model, the dipole-induced-dipole model, and the analytical model^27,28^. In the case of dipole–dipole interaction between two similar spherical droplets aligned with the applied electric field, the following electrostatic force can be derived^29^.

2 $${F}_{dip}=\frac{-12\pi {\omega }^{2}{\varepsilon }_{c}{E}^{2}{r}_{1}^{3}{r}_{2}^{3}}{{s}^{4}}\left(3K-1\right),$$

3 $$\omega =\frac{{\varepsilon }_{d}-{\varepsilon }_{c}}{{\varepsilon }_{d}+2{\varepsilon }_{c}},$$

Clausius–Mossotti factor (ω) is defined in Eq. (3). The coefficient K can be written as shown in Eq. (4)^28^.

4 $$K=1+\frac{\omega {r}_{1}^{3}{s}^{5}}{{\left({s}^{2}-{r}_{2}^{2}\right)}^{4}}+\frac{\omega {r}_{2}^{3}{s}^{5}}{{\left({s}^{2}-{r}_{1}^{2}\right)}^{4}}+\frac{3{\omega }^{2}{r}_{1}^{3}{r}_{2}^{3}\left(3{s}^{2}-{r}_{1}^{2}-{r}_{2}^{2}\right)}{{\left({s}^{2}-{r}_{1}^{2}-{r}_{2}^{2}\right)}^{4}}.$$

**Figure 2:**
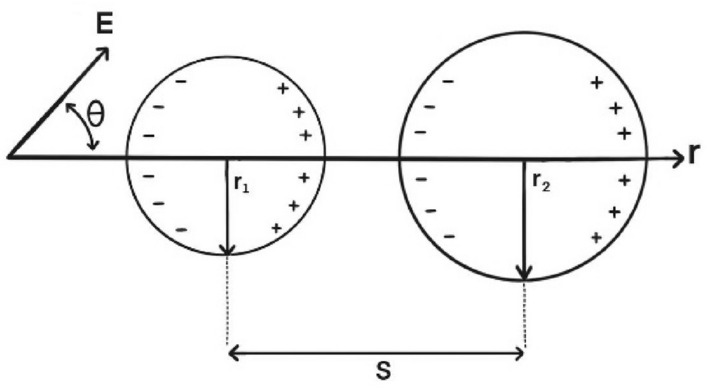
Interaction of two drops in an electric field (modified after Mhatre et al.^52^).

As shown in Fig. 2, s is the separation distance, and r_1_ and r_2_ are droplet radii. E is the electric field strength, and ε_c_ and ε_d_ are permittivities of oil and water respectively.

### Electrophoretic force

Electrophoretic forces refer to both attractive and repulsive forces that occur when charged droplets interact with electrodes within a consistent voltage field. These forces depend on the strength of the electric field, the size of the droplets, and the conductivity of the surrounding oil. The relationship developed by Lundgaard et al.^30^ can be written as follows.

5 $${F}_{ele}=0.67{\varepsilon }_{c}{\pi }^{3}{r}^{2}{E}^{2}.$$

### Dielectrophoretic force

Dielectrophoresis can be generally defined as the behavior where droplets, such as aqueous droplets in oil, with higher permittivity than the surrounding medium, tend to migrate towards regions of higher electric field intensity. The average dielectrophoretic force acting on an individual droplet in a non-uniform external electric field can be estimated by considering dipole effects as written in Eq. (6)^31^.

6 $${F}_{diele}=2\pi {r}^{3}{\varepsilon }_{c}\beta \nabla \left(\overrightarrow{E}.\overrightarrow{E}\right)=2\pi {r}^{3}{\varepsilon }_{c}\beta \nabla {|\overrightarrow{E}|}^{2},$$

## Modelling approach

Industrial processes commonly involve dispersed two-phase flows, which are characterized by the presence of small droplets or particles suspended within a continuous phase. However, relying solely on models that consider a single droplet size is inadequate for accurately describing the behavior of dispersed phases within the continuous phase^32^. To address this limitation, the population balance model has been utilized to study the unique characteristics of dispersed flow processes, including crystallization, liquid–liquid extraction, solid–liquid leaching, and water–oil emulsion separation. The population balance equations (PBE) provide a macroscopic-level understanding of how the particle number density changes over time^33^.

We study the modelling of a dispersed phase system (liquid–liquid) within the context of inline electrostatic coalescence and simplify the general PBE^33,34^ to obtain the zero-dimensional, time-dependent form of the equation. Equation (7) assumes that the spatial distribution of droplets is random and homogeneous. Two main aspects of the solution of the PBE should be taken into account: (i) discretization of PBE, (ii) closures of PBE.

7 $$\frac{df}{dt}={\int }_{\upsilon }^{\infty }{r}^{B}\left(\upsilon ,\widetilde{\upsilon }\right)f\left(\widetilde{\upsilon }\right)d\widetilde{\upsilon }-\frac{f\left(\upsilon \right)}{\upsilon }{\int }_{0}^{\upsilon }\widetilde{\upsilon }{r}^{B}\left(\widetilde{\upsilon },\upsilon \right)d\widetilde{\upsilon }+ \frac{1}{2}{\int }_{0}^{\upsilon }{r}^{C}\left(\widetilde{\upsilon },\upsilon -\widetilde{\upsilon }\right)f\left(\widetilde{\upsilon }\right)f\left(\upsilon -\widetilde{\upsilon }\right)d\widetilde{\upsilon }-f\left(\upsilon \right){\int }_{0}^{\infty }{r}^{C}\left(\widetilde{\upsilon },\upsilon \right)f\left(\widetilde{\upsilon }\right)d\widetilde{\upsilon },$$

where f is the number density probability function, and r^C^ and r^B^ respectively, are the breakage and coalescence kernels. The right-hand side of the Eq. (7) is the source and sink terms of the transport problem in the external coordinate (time) due to the transport of droplets in the internal coordinate (size).

The availability of analytical solutions for the population balance equation is limited and usually requires specific closures and initial size distributions^33^. However, we focus on scenarios where analytical solutions are not readily available. Therefore, numerical techniques must be employed to discretize the PBE in both the internal coordinate and time domains.

### Discretization in the internal coordinate

In order to distinguish droplets as the secondary phase, concerning their properties of interest, discretization is performed in the internal coordinate. Internal coordinates can be related as in the case of the size and the volume of spherical droplets, where knowing one of these variables is sufficient to determine the other properties. The choice of internal coordinate variables highly depends on the specific application and imposes constraints on the available numerical methods. When considering the discretization approach, two factors are crucial: (i) the types of population dynamics involved, such as coalescence and breakage of droplets in our case, and (ii) the specific properties of interest included in the particle state vector. These considerations guide the selection of an appropriate numerical method^35^.

We applied the “method of classes” to discretize the PBE within the internal coordinate, which corresponds to the size of droplets. This method involves treating each “class” as a distinct size range. We define zero-order methods as approaches that approximate the continuous particle size distribution by utilizing a set of linearly independent functions with zero-order characteristics^36,37^. The discretized form of the PBE for the probability of the number density of the i-th class, denoted as f_i_ (where i, j, and k are class indices), can be expressed as Eq. (8):

8 $$\frac{\partial {f}_{i}}{\partial t}={\sum }_{j=i}^{n}{r}_{i,j}^{B}{f}_{i}\Delta {\upsilon }_{j}-\frac{{f}_{i}}{{\upsilon }_{i}}{\sum }_{j=1}^{i}{\upsilon }_{j}{r}_{j,i}^{B}\Delta {\upsilon }_{j}+\frac{1}{2}{\sum }_{j=1}^{i}{r}_{j,k}^{C}{f}_{i}{f}_{k}\Delta {\upsilon }_{j}-{f}_{i}{\sum }_{j=1}^{n}{r}_{j,i}^{C}{f}_{j}\Delta {\upsilon }_{j}.$$

$$fori=\mathrm{1,2},\dots ,n$$

### Discretization in the external coordinate

Numerical studies have mainly focused on the solution of the PBE developing approaches to discretize the internal coordinate. However, Bakhbakhi^38^ investigated the numerical solution of the PBE in a zero-dimensional (0D) context, which allows for exploring different numerical techniques for discretizing the external coordinate. Equation (9) shows a PBE with implicitly written right-hand side utilizing the one-step-θ scheme.

9 $$\frac{{f}^{n+1}-{f}^{n}}{\Delta t}=\theta {S}^{n+1}+\left(1-\theta \right){S}^{n},$$

where the time index is represented by the superscript “n”, and the parameter θ corresponds to the time-stepping scheme. Specific values of the θ parameter correspond to commonly known schemes, such as θ = 0 for the Forward Euler method, θ = 1 for the Backward Euler method, and θ = 0.5 for the Crank-Nicolson scheme. Therefore, the resulting equation in its fully discretized form can be expressed using Eq. (10).

**Figure 3:**
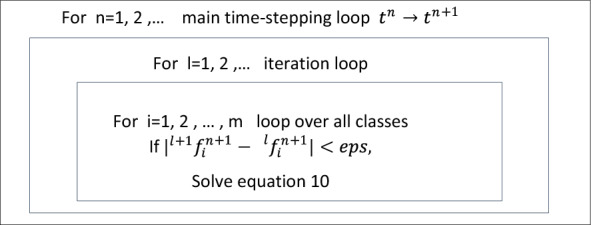
Solution of a discrete population balanced equations^49^.

Figure 3 illustrates the algorithm that we utilized to solve this nonlinear equation.

10 $$\frac{{f}^{n+1}\left({\upsilon }_{i}\right)-{f}^{n}\left({\upsilon }_{i}\right)}{\Delta t}=\theta S\left({f}^{n+1}\left({\upsilon }_{i}\right),{f}^{n+1}\left({\upsilon }_{j}\right)\right)+\left(1-\theta \right)S\left({f}^{n}\left({\upsilon }_{i}\right),{f}^{n}\left({\upsilon }_{j}\right)\right)$$

### Closures of population balance equations

The PBE for dispersed liquid–liquid systems involves breakage and coalescence kernels (r^B^ and r^C^, respectively), which describe all the important physical aspects associated with breakup and coalescence phenomena. While it is possible to incorporate coalescence kernels into a unified framework, the behaviour of breakage kernels can exhibit significant variations^39^, which adds complexity to employing the appropriate breakage kernel for this study.

The electro-coalescence process in the new device can be divided into two distinct stages. In the initial stage, droplets approach each other, and the shearing forces within the emulsion play a critical role. In the second stage, the extremely close droplets rapidly merge as a result of the electric field, leading to a significant decrease in coalescence time^40^. Other factors are the potential deformations that may occur within the droplets. If the strength of the electric field exceeds a certain threshold, the droplets may undergo deformation or even break, leading to electric dispersion^27,30^. Atten^3^ derived the expression for the critical electric field, E_c_, which represents the threshold value that causes droplets to break:

11 $${E}_{c}=0.64{\left(\frac{\sigma }{{2\varepsilon }_{c}d}\right)}^{0.5}.$$

E_c_ is determined by the surface tension (σ), oil permittivity (Ɛ_c_), and droplet radius (d). When the electric field exceeds E_c_, the interface becomes unstable, resulting in dispersion and the formation of significantly smaller droplets. For instance, with an electric field of 4 kV/cm, such dispersion occurs only for droplets larger than approximately 5 mm in diameter. However, since the coalescence of the emulsions under investigation seldom yields such large droplets, the dispersion phenomenon is considered insignificant within the scope of this study. Nevertheless, it is essential to consider the deformation of the interface when examining the interaction between closely positioned droplets.

### Coalescence kernel

Waterman^41^ suggested that the fundamental mechanism involved in the electro-coalescence phenomenon is the dipolar force between neighbouring droplets that is resulting from the interaction of dipoles induced by the electric field. This process is known as “dipolar coalescence”. Panchenkov et al.^42^ derived a formula for coalescence kernel in the scenario of dipolar coalescence involving submicronic droplets. Their approximate expression was obtained by comparing the influence of electric forces with the thermal energy kT. Building upon this foundation, Atten^3^ derived an approximate expression for the effective force, Eq. (13), assuming that the potential difference between two spheres i and j is ΔV = (r_i_ + r_j_ + s) EcosΘ, where Θ represents the angle between the electric field vector and the line connecting the centers of the two droplets.

12 $${r}^{C}={\alpha K}^{C},$$

13 $$F=\pi \varepsilon {E}^{2}{cos}^{2}\Theta {\left(a+b+s\right)}^{2}/\left[s\left(2s+b+{b}^{2}/a\right)\right].$$

Continuing from the framework established in Eqs. (13) and (2) and relaxing Atten's simplifying assumption that the distance between droplets is much smaller than the smaller droplet, our model enables more detailed calculations and a comprehensive understanding of droplet behavior in electro-coalescence. However, we still account for the case where droplets are in direct contact (s = 0) at the end of our calculations.

For two colliding drops, because the drops are touching each other at specific points, and their boundaries are in direct contact, we can consider that there is zero separation and there is a geometrical contact. By using the law relative to a sphere and a plane interface^43^ we can derive the collision rate affected by electric field as follows:

14 $${K}_{1}^{c}\left({r}_{i},{r}_{j}\right)=\frac{{\varepsilon E}^{2}{\left({r}_{i}+{r}_{j}\right)}^{2}}{6{\mu }_{o}\left({r}_{j}+{{r}_{j}}^{2}/{r}_{i}\right)},$$

where K (r_i_, r_j_) is the expression of collision rate between droplets of radii r_i_ and r_j_ (r_i <_ r_j_).

So far, our focus has been on the impact of electric forces in facilitating contact between water droplets. Based on our calculations using Eq. (14) and the results of performance tests conducted on the inline electro-coalescer^13,44^, we can conclude that the observed effectiveness of this innovative device cannot be solely attributed to electric forces. This is because in the presence of shear, the emulsion experiences relative motion of dispersed droplets, resulting in numerous collisions.

A theoretical examination of geometric collisions within a uniform shear flow was initially conducted by Smoluchowski^45^, assuming rectilinear trajectories for droplets. Combining that with the concept of orthokinetic coalescence efficiency, introduced by Mousa and Ven^46^, we can assume that the hydrodynamic interactions and the colloidal forces acting on the droplets are negligible except when they come into contact, and arrive at the following generalized equation:

15 $${K}_{2}^{c}\left({r}_{i},{r}_{j}\right)=1.33{{\sf T}\kern-7.5pt{}_{\boldsymbol{\upiota}}}\,\,{\left({r}_{i}+{r}_{j}\right)}^{3},$$

where ${\sf T}\kern-7.5pt{}_{\boldsymbol{\upiota}}$ represents the shear rate. This relation implies that every collision leads to coalescence of the colliding droplets. Therefore, a coalescence efficiency (α) function needed to be introduced to represent the proportion of collisions that lead to coalescence. If all collisions result in coalescence, α = 1. However, based on the majority of experimental findings in the literature, α is significantly less than 1^47^. Additionally, the efficiency values are influenced by the sizes of the colliding drops. Herein, Eq. (16) for coalescence efficiency, as proposed by Nandi et al.^48^ was employed in the population balance equation.

16 $$\alpha \left(i,j\right)={\alpha }_{0}{\left[\frac{4\frac{{r}_{i}}{{r}_{j}}}{{\left(1+\frac{{r}_{i}}{{r}_{j}}\right)}^{2}}\right]}^{C}{\left[1+{\left(1-\frac{{r}_{i}}{{r}_{c}}\right)}^{2}\right]}^{-m}{\left[1+{\left(1-\frac{{r}_{j}}{{r}_{c}}\right)}^{2}\right]}^{-m},$$

where α_0_ is coalescence efficiency constant, r_i_ and r_j_ are radius of colliding drops and r_c_ is the critical radius of drops at which the coalescence efficiency is at its maximum or minimum. The parameters of the model (α_0_, C, m, r_c_) are constants obtained by a regression procedure applied to the experimental droplet size distributions, such that the population balance model predicts the experimental results.

### Breakage kernel

When investigating droplet breakage, it is important to consider that breakage is not the dominant phenomenon in the system. Considering the configuration of the inline electro-coalescer, the breakage of droplets is primarily controlled by moderately turbulent flow rather than the electric field. This assertion is supported by the assumption that the strength of the electric field is kept lower than Ec. Calculations based on the Reynolds number, within the range of operational conditions, suggest the flow can be characterized as moderately turbulent.

The flow causes deformation and stretching of the droplets in one direction, leading to the formation of a necking region. This necking region further contracts, ultimately resulting in the breakage of the droplets. In order to calculate the droplet-size distribution, it is necessary to specify the frequency of droplet breakups. Additionally, the size distribution of the daughter droplets formed during the breakage process needs to be known. We made the following assumption regarding the breakup kernel function.

1. Only the binary breakage is considered.
2. The breakup of a droplet is governed by the balance between the interfacial force acting on the droplet surface and the inertial force of the colliding eddy. The interfacial forces are influenced by the shape of the droplet and the sizes of the resulting droplet fragments^34,49^*.*
3. Only eddies with length scales smaller than the droplet diameters can induce breakage. Larger eddies lead to droplet transport instead. For the same reason, the length scale of the eddy has to be larger than the diameter of the smaller resulting droplet fragment^34,39^.

We utilized Lehr^50^ concept of breakage kernels in turbulent systems as a basis for modelling the breakup behaviour in the liquid–liquid system under moderately turbulent conditions. Lehr provided a framework for characterizing particle breakage, Eq. (17), where the collision frequency was derived analogously to the kinetic gas theory. We applied and extended this concept to our specific liquid–liquid system, with the objective of capturing the dynamics of breakup phenomena in our modelling approach.

We assumed the formation of two equal fragments upon breakup. To account for the assumption, we incorporated the daughter size distribution (DSD) denoted as β(i, j), Eq. (18), in the main equation, Eq. (8). The DSD allowed us to describe the distribution of fragment sizes resulting from the breakup process. The breakage kernel is defined as the multiplication of the total breakage rate, K^B^, and daughter size distribution, $\beta$.

17 $${r}^{B}={\beta K}^{B},$$

18 $${K}^{B}=\frac{{d}^{*5/3}}{2T}exp\left(-\frac{\sqrt{2}}{{{d}^{*}}^{3}}\right),$$

$$L={\left(\frac{\sigma }{{\rho }_{d}}\right)}^{0.6}{\varepsilon }^{-0.4},$$

$$T={\left(\frac{\sigma }{{\rho }_{d}}\right)}^{0.4}{\varepsilon }^{-0.6},$$

$${d}^{*}=d/L.$$

By integrating the concept of breakage kernels and incorporating the assumption of equal fragment formation, we are able to study the collision frequency and subsequent breakup dynamics, which leads to understanding the complex connection between turbulence and the breaking apart of droplets.

19 $$j\,even:\beta \left( {i,j} \right) = \left\{ {\begin{array}{*{20}c} 2 & {i = \frac{j}{2}} \\ 0 & {otherwise} \\ \end{array} } \right.$$

$$j\,odd:\beta \left( {i,j} \right) = \left\{ {\begin{array}{*{20}c} 1 & {i = \frac{{j - 1}}{2},\frac{{j + 1}}{2}} \\ 0 & {otherwise} \\ \end{array} } \right.$$

The calculations are refined to include both the parameters of the maximum stable (critical) diameter, as stated in Eq. (20), and the minimal diameter of the daughter drop as described in Eq. (21) by Zaccone et al.^51^.

20 $${d}_{c}={\left(\frac{12\sigma }{\beta \rho }\right)}^{0.6}{\varepsilon }^{-0.4},$$

21 $${d}_{pmin}={\left(\frac{12\sigma }{\beta \rho }\right)}^{1.5}{\varepsilon }^{-1}.$$

## Results and discussion

### Model validation

**Figure 4:**
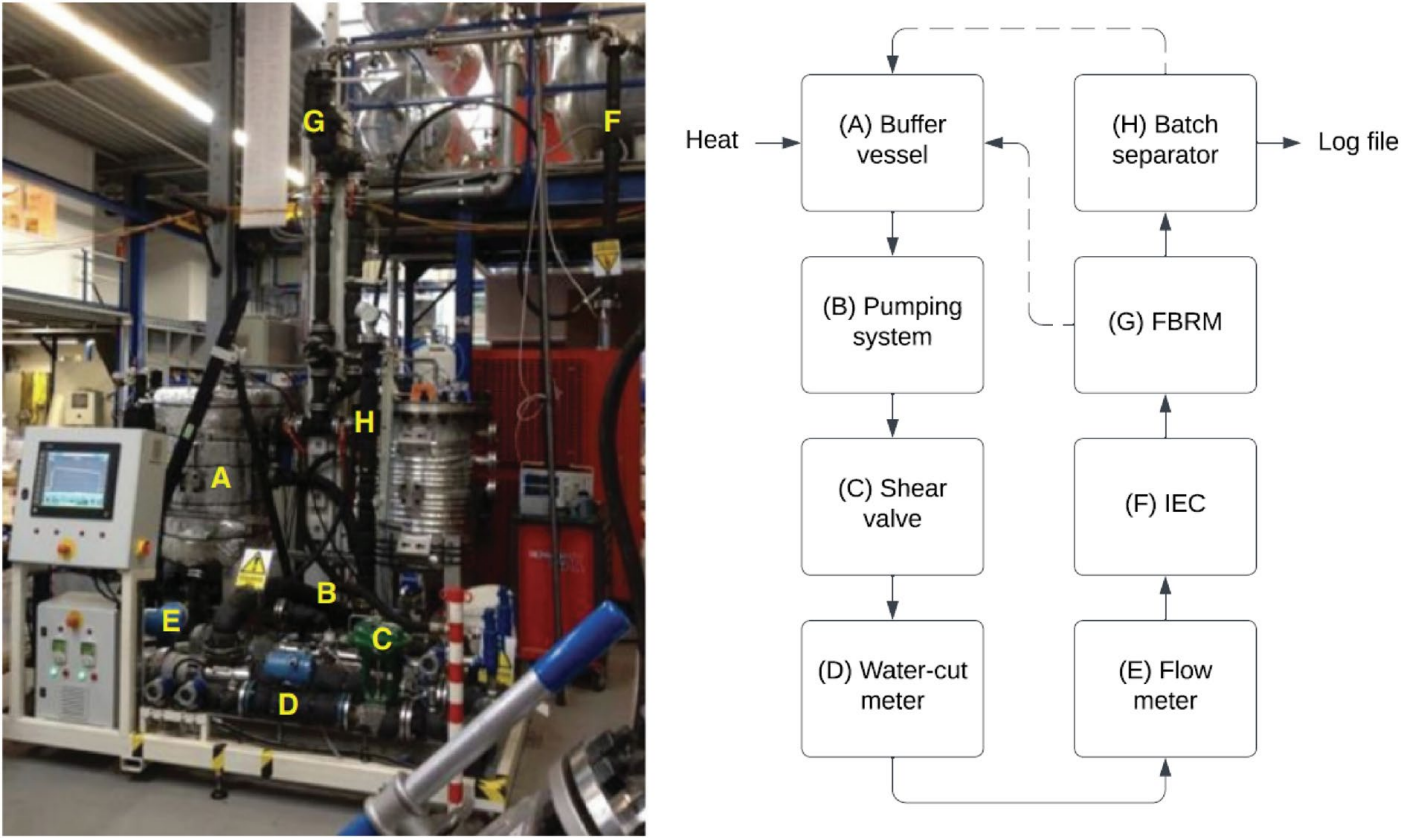
Image of experimental setup with highlighted main components (left) and a simplified process flow diagram of the experiment (right)^13^.

We used the experimental data of the inline device’s performance tests conducted by Grave et al.^13^ and Westra et al.^44^ as the basis to validate our modelling approach. Both studies employed similar experimental setups, which included a compact closed loop to investigate the electro-coalescence phenomenon of dispersion of water droplets in crude oil under continuous flow conditions. As illustrated in Fig. 4 the experimental setup commenced with a buffer vessel housing the fluids. This was followed by a pumping system, where a shear valve continuously sheared the emulsion to generate a large number of droplets. Subsequently, a water-cut meter and a flowmeter were employed, in conjunction with an IEC device. The setup also included a focused beam reflectance measurement probe (FBRM) to monitor droplet size, and it completed with a batch separator at the end of the process. The dual heating system connected to the buffer vessel ensured that the emulsion was heated, and a constant temperature was maintained during the test. The FBRM probe, an advanced in-situ droplet size monitoring tool, was positioned after the IEC to assess the impact of the device on the distribution of water droplet sizes by measuring the size of water droplets as they flowed through the pipe. This real-time measurement offered valuable insights into the rate of coalescence and breakage of droplets under different operating conditions. This offered a direct assessment of the coalescence efficiency, which directly correlated with the water separation efficiency in the downstream separator.

The required experimental properties to solve the developed mathematical model, such as specifications of the crude oil, water, and electrostatic device, can be found in Table 1.

**Table 1:** Fluid properties and electrostatic device specification^13,44^.

Property	Value
Medium crude oil density (kg/m^3^)	865
Medium crude oil viscosity (cP)	13
Heavy crude oil density (kg/m^3^)	905
Heavy crude oil viscosity (cP)	38
Water density (kg/m^3^)	998
Water viscosity (cP)	1
Water cut (%)	30
Device length (inch)	4
Voltage (kV)	0, 1, 2, 3, 4
Upstream pressure drop (bar)	0.25
Flow rate (m^3^/h)	5, 10, 15, 20, 25

**Figure 5:**
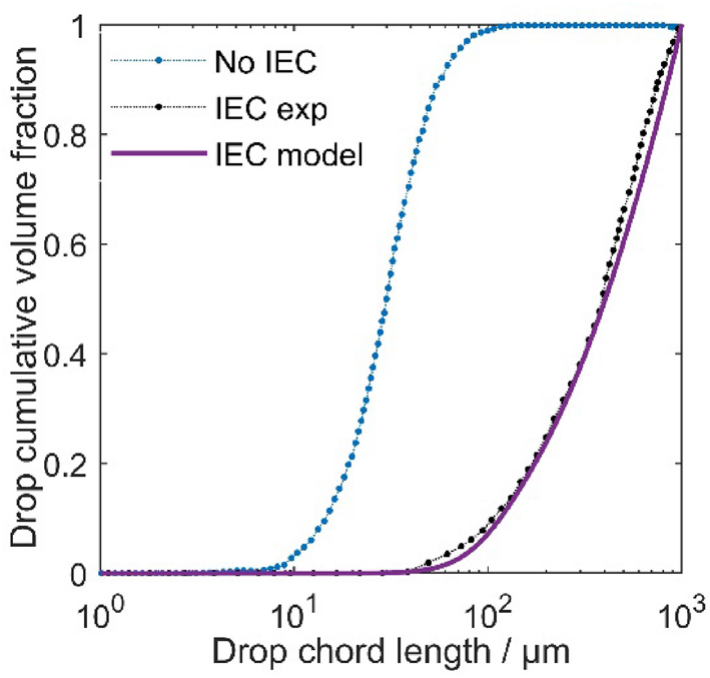
Comparison of modelling and experimental values of cumulative volume fractions as the representation of droplet size distribution at the outlet of IEC.

Figure 5 illustrates the size distribution and the spatial arrangement of droplets in two scenarios: (i) the utilization of an electric field (IEC switched on) and (ii) without it (IEC switched off). The distribution is represented by plotting the cumulative volume fraction of droplets against their cord length at the coalescer device outlet. The chord length of a droplet can be representative of droplet diameters, as the chord length is the shortest distance between two points on the droplet's circumference and passes through the droplet's centre.

Both scenarios exhibit smooth curves without sudden jumps, indicating a uniform size distribution of droplets. This means that droplets are distributed relatively evenly without significant clustering or localized variations.

In the scenario without the presence of an electric field, the analysis of the cumulative volume fraction reveals a distinctive trend. Initially, as the droplet size increases from 10 μm, the cumulative volume fraction sharply increases until reaching approximately 50 μm, where it approaches a plateau at the cumulative volume fraction equal to one. This indicates that all of the droplets in this size range have been taken into account, and there are no further larger droplets present in the system. Another parameter is the relevant chord length of 0.5 volume fraction, D_50_, which represents the median droplet size of the system. Analysing this parameter is important because it provides a value that allows for a direct comparison of droplet sizes between different scenarios. The D_50_ value for the scenario without using electricity is approximately 30 μm. This indicates that 50% of the droplet volume is contained within droplets smaller than or equal to 30 μm in chord length.

When comparing the scenario with the electric field to the one without it, a noticeable change in the chord length range is evident. In the electric field scenario, the cumulative volume fraction curve begins to rise at around 30 μm and reaches one at a chord length of 1000 μm, with a D_50_ value equal to approximately 300 μm. This shift indicates a significant change in the droplet size distribution, emphasizing the prevalence of larger droplets in the scenario with the electric field compared to the scenario without it. Therefore, we conclude that the presence of the electric field has a substantial impact on the distribution of droplet sizes, ultimately enhancing the efficiency of water removal. The cumulative droplet size distribution obtained by the model is consistent with experimental results.

### Parametric analysis of model characteristics

**Figure 6:**
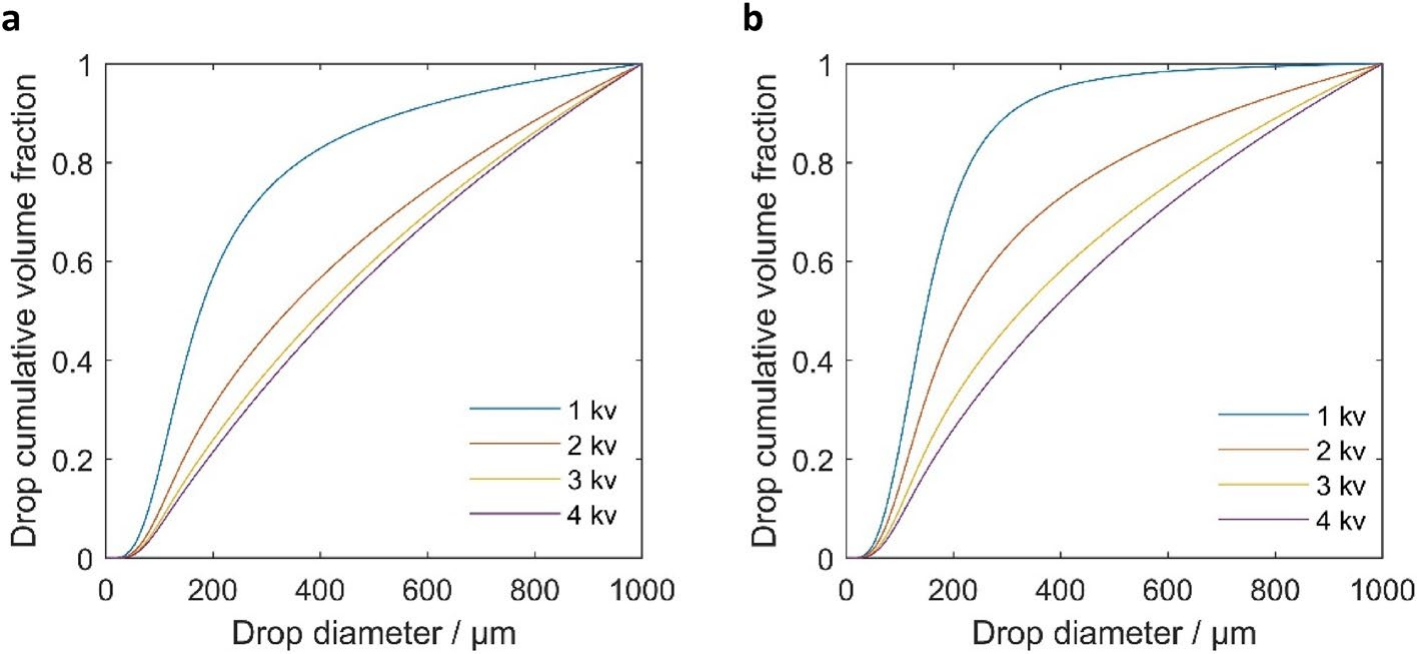
Droplet size distribution for (**a**) medium and (**b**) heavy oil at the outlet of IEC with different voltages.

The strength of the electric field plays a significant role in the crude oil dehydration process as it affects the efficiency of water separation. Figure 6 illustrates the droplet size distributions at the outlet of the electro-coalescer device at the flow rate of 10 m/s for two oil types of medium and heavy. The effects of various voltages (1, 2, 3 and 4 kV) were investigated using the proposed model. As the electric field causes water droplets dispersed in the oil phase to become polarized and collide, the increase in voltage intensifies the polarization effect, leading to a stronger-range attraction force among the droplets. This increased force facilitates the disruption of the interfacial film between them, promoting their merging and the formation of larger droplets. Therefore, the increase in electric field strength causes the droplet size distributions to shift towards larger sizes, indicating that the dominant interaction of droplets is coalescence in the coupling device at different voltages. The coalescence effect is enhanced significantly with the increase in voltage. However, when the voltage is increased from 3 to 4 kV for medium oil, the cumulative size distribution curve of droplets is coincident, thereby indicating that coalescence cannot be continuously improved by raising the voltage.

The results from the two plots indicate that heavy oil requires higher voltage levels to achieve similar coalescence effects and water separation efficiency as compared to medium oil. As seen in the droplet size distributions, heavy oil exhibits smaller median droplet diameters (D_50_) at the same voltage levels compared to medium oil. In our case, at 2 kV, heavy oil's D_50_ is approximately 220 µm, while medium oil's D_50_ is around 340 µm. The D_50_ mentioned here represents the average particle size of droplets which is the diameter corresponding to the droplets cumulative volume fraction of 0.5.

**Figure 7:**
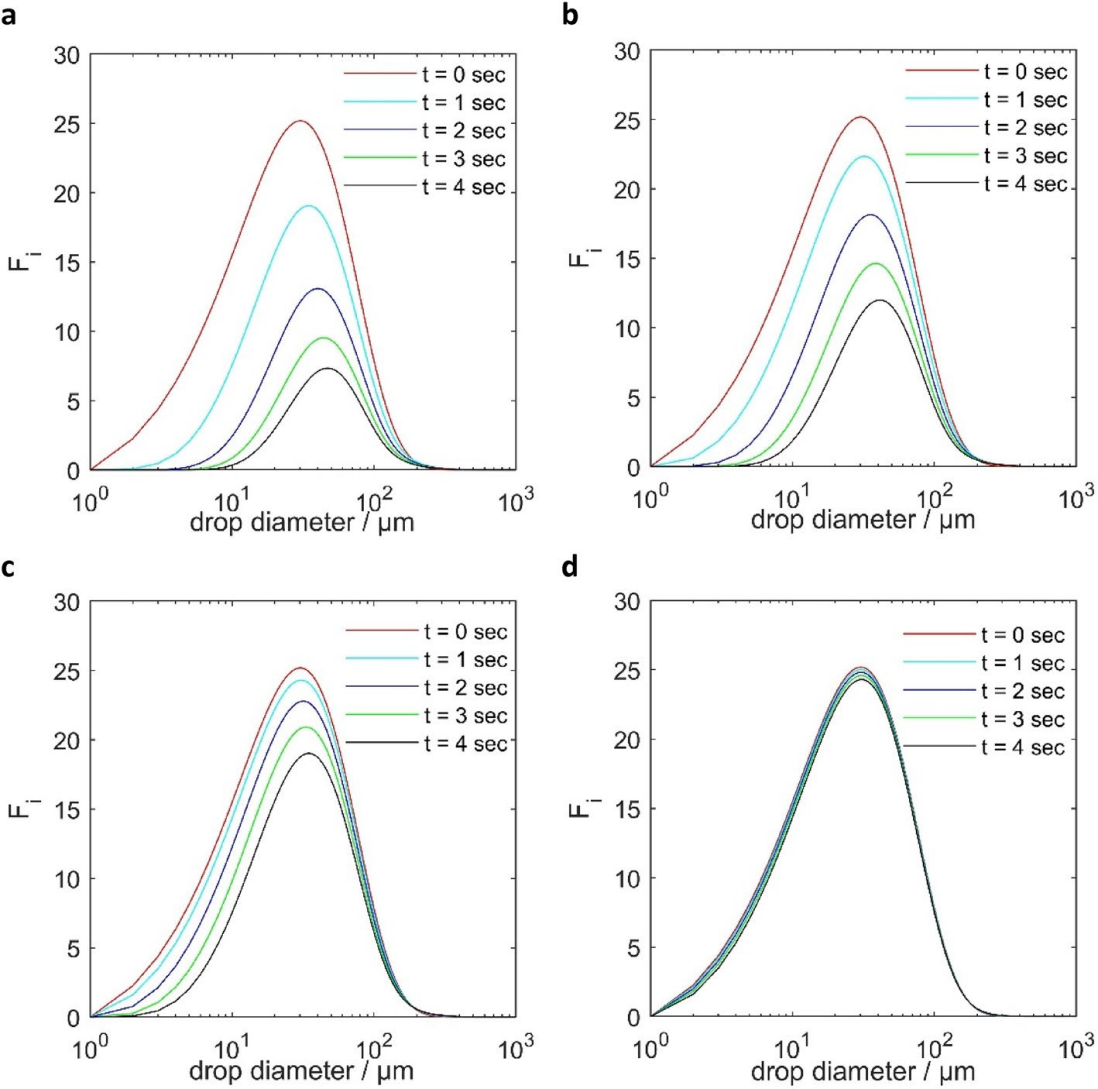
Temporal changes of droplet size distribution in IEC with different electric field strengths: (**a**) 4kv, (**b**) 3kv, (**c**) 2kv, (**d**) 1kv, medium oil.

**Figure 8:**
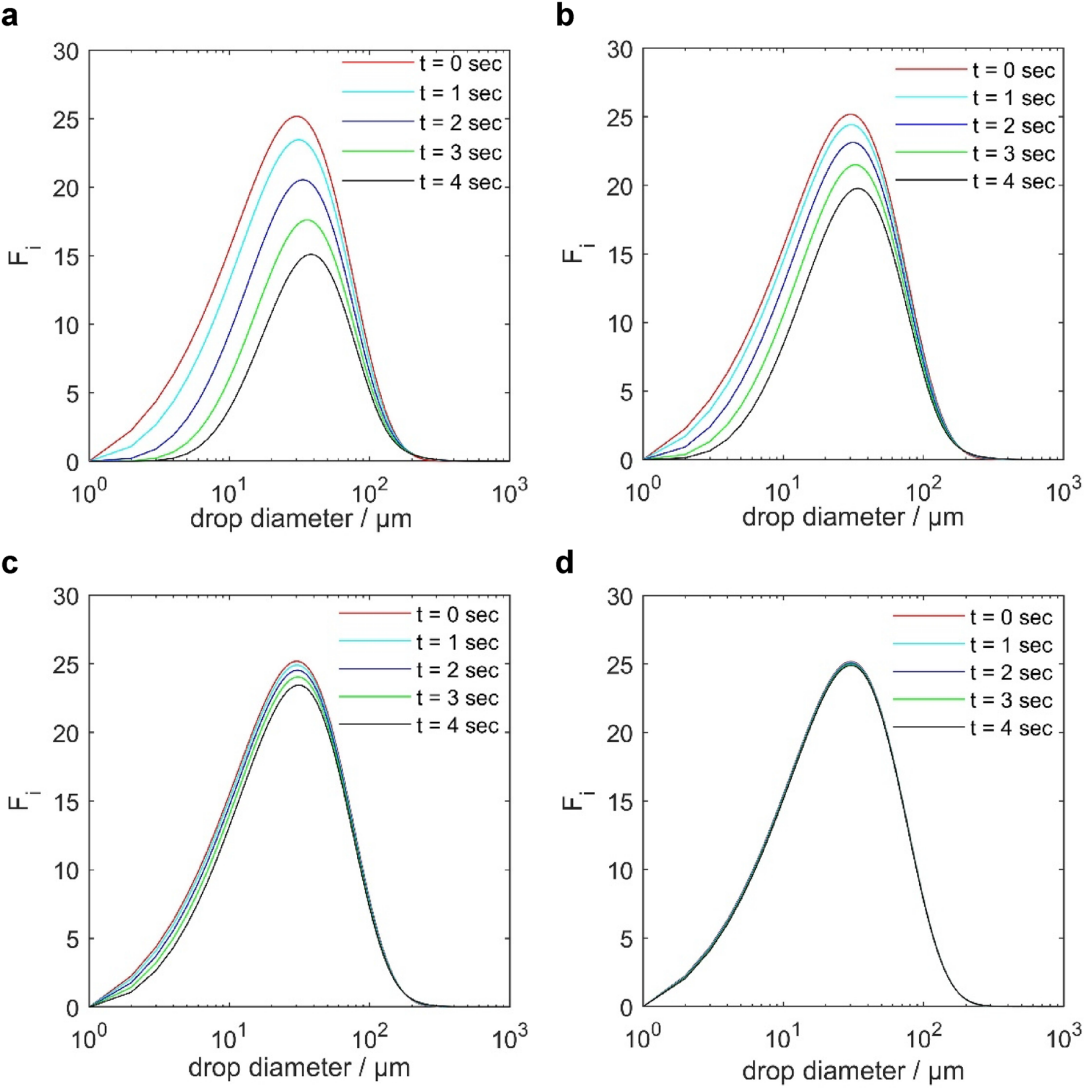
Temporal changes of droplet size distribution in IEC with different electric field strengths: (**a**) 4kv, (**b**) 3kv, (**c**) 2kv, (**d**) 1kv, heavy oil.

In our study, a gravitational vessel is considered to be utilized downstream of IEC to evaluate the water separation. Following Aryafard et al.^20^, droplets with diameters exceeding 294 µm were considered to be effectively separated in a downstream gravitational vessel. Figures 7 and 8 show the temporal evolution of the droplet size distribution over a wide range of droplet diameters (1 to 1000 µm) for two different oils. The results are based on the number density probability function for different droplet diameters, representing the number of water droplets per unit volume, at various residence times (t = 0, 1, 2, 3, and 4 s).

The curves follow a normal distribution, exhibiting a characteristic bell-shaped pattern, which features a prominent peak at the centre and a gradual decline in droplet counts towards the outer edges. This pattern implies a tendency for specific droplet sizes to become more prevalent, while less frequent sizes occur towards the extremes of the distribution.

The plots demonstrate a complex behaviour over time, considering both coalescence and breakage processes. Coalescence is observed as the dominant mechanism, leading to the merging of smaller water droplets into larger ones, which is evident from the reduction in the peaks of the number density probability function and the reduction of the overall number of droplets. Simultaneously, the rightward shift in the plots indicates an increase in droplet size over time. Note that these observations pertain to droplets smaller than 294 µm.

While the peak decrease and the rightward shift are apparent across all voltage levels, higher voltages are observed to promote a more significant coalescence effect, leading to a considerable decrease in the peak number density of smaller droplets and a more pronounced shift towards larger droplet sizes over time.

**Figure 9:**
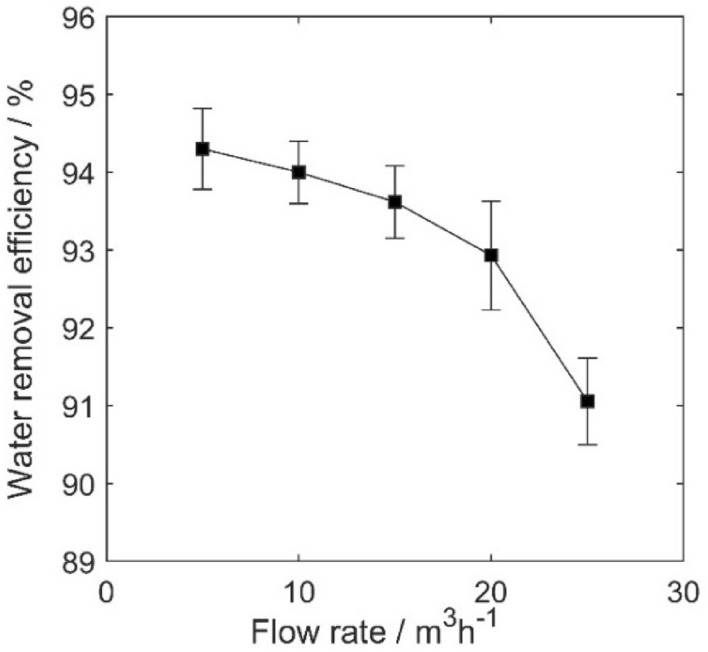
Assessing water removal efficiency in a 4 kV IEC system with medium Oil: an analysis of flow rate variations and modeling error.

Figure 9 illustrates the relationship between the water removal efficiency and the inlet flow rate in an IEC operating at a fixed voltage of 4 kV for medium oil. The study examines flow rates ranging from 25 to 5 m^3^/h with 5 m^3^/h intervals. The graph illustrates that with decreasing flow rates, the water removal efficiency of the system increases. However, it is noteworthy that as the flow rate continues to decrease, the efficiency gain becomes less substantial, with gains of 2%, 0.7%, 0.4%, and 0.3% with each 5 m^3^/h flow rate reduction, respectively. Therefore, further reductions in flow rate are not advisable in the context of industrial operations. This trend aligns with experimental observations^13,44^. We can observe from this figure that the modeling error remains minor over the entire range of flow rates.

The result highlights that optimizing the flow rate within the constraints of industrial operational conditions can yield a water removal efficiency of up to 94.3%. Compared to Figs. 6, 7, 8, the efficiency of electro-coalescence in this system is more responsive to adjustments in the electric field strength and the residence time of the fluid within the pipeline, compared to adjustments in the flow rate.

## Conclusions

In this study, a population balance model, combined with coalescence and breakage kernels is developed to predict the evolving droplet size distribution and water separation behaviour in modern inline electrostatic coalescers (IEC) in emulsified oil systems. The significance of the model lies in its ability to address not only the predominant coalescence of droplets but also the crucial factor of emulsion droplet breakage, which directly influences the efficiency of the water separation process.

The cumulative droplet size distribution and separation efficiency obtained by the model are consistent with the experimental results presented in literature^13,44^. The modelling deviations were shown to be minor. Therefore, the model presented can serve as a valuable tool for improving the performance of inline electrostatic coalescers and advancing their practical applications in the petroleum industry.

The effects of electric field strength, residence time and inlet flow rate on droplet size distribution in the device are investigated using numerical methods. The results show that the application of a high-voltage electric field effectively promotes the coalescence of small droplets. The mean droplet diameter and separation efficiency of droplets initially increase noticeably and then do not change much with an increase in voltage. When the voltage is 4 kV, and the flow rate is 5 m^3^/h, after 4 s, the water removal efficiency reaches 94.3% and the mean diameter of droplets increases from 30 µm at the beginning to 432 µm at the outlet of the device. As the inlet flow rate decreases, the coalescence effect of droplets and D_50_ gradually increases. Separation efficiency initially experiences a significant increase but the rate of improvement slows down when further decreasing the flow rate. The efficiency of electro-coalescence in this system is more responsive to adjustments in the electric field strength and the residence time of the fluid within the pipeline, compared to adjustments in the flow rate.

## Data Availability

The datasets generated during and/or analysed during the current study are available from the corresponding author on reasonable request.

## List of symbols

${d}_{stokes}$: Stokes diameter, µm
$d$: Droplet diameter, µm
${\mu }_{c}$: Dynamic viscosity of the continuous phase, P
${u}_{s}$: Relative velocity of the dispersed droplets through the continuous phase, cm.s^–1^
$g$: Constant of gravitational acceleration, cm.s^–2^
${\rho }_{d}$: Density of the dispersed phase, g.cm^–3^
${\rho }_{c}$: Density of the continuous phase, g.cm^–3^
$f$: Number density probability function
$$\sigma$$: Interfacial tension, N.m^–1^
$${r}^{B}$$: Breakage kernel
$${r}^{C}$$: Coalescence kernel
$\upsilon$: Volume of droplet, µm^3^
${\varepsilon }_{c}$: Relative permittivity of continuous phase, F·m^–1^
${\varepsilon }_{d}$: Relative permittivity of dispersed phase, F·m^–1^
$E$: Electric field intensity, kV.cm^–1^
${E}_{c}$: Critical electric field intensity, kV.cm^–1^
$r$: Droplet radii, µm
$$K$$: A constant
$s$: Drops separation distance, µm
θ: Time-stepping scheme parameter
Θ: Angle between the electric field vector and the line connecting the centres of two droplets
t: Time, sec
ω: Clausius–Mossotti factor

## Subscripts

$$c$$: Continuous phase
$$d$$: Dispersed phase
i, j, k: Class indices

## Superscript

n: Time index
C: Coalescence
B: Breakage
